# Online Survey of Medical and Psychological Professionals on Structured Instruments for the Assessment of Work Ability in Psychiatric Patients

**DOI:** 10.3389/fpsyt.2018.00453

**Published:** 2018-09-25

**Authors:** Roman Schleifer, Alex Gamma, Ingeborg Warnke, Mounira Jabat, Wulf Rössler, Michael Liebrenz

**Affiliations:** ^1^Institute of Forensic Medicine, Department of Forensic Psychiatry, University of Bern, Bern, Switzerland; ^2^Department of Psychiatry, Psychotherapy and Psychosomatics, Psychiatric Hospital, University of Zurich, Zurich, Switzerland

**Keywords:** work ability, assessment, psychiatry, rehabilitation, online survey

## Abstract

**Objective:** To evaluate perceived needs and difficulties related to instruments for assessing work ability in individuals with mental disorders.

**Method:** We conducted an online survey of 104 German-speaking medico-legal experts (forensic psychiatric and psychology experts, insurance physicians) and therapists.

**Results:** The large majority of respondents reported they would welcome a standardized, structured instrument for the assessment of work ability. High predictiveness, inter-rater agreement, comprehensibility for laymen, and symptom validity were desired in roughly equal measure as the main characteristic of such an instrument. More women than men, and more medico-legal experts than therapists, considered symptom validation as always necessary. Pain, personality, and affective disorders were perceived to be the most difficult disorders in the context of work ability assessments.

**Conclusion:** Our survey documents professionals' wish for a structured assessment of work ability in both medico-legal and therapeutic settings.

## Introduction

In the past decades, most OECD member states have seen a substantial rise in spending on disability benefits, with mental disorders accounting for at least 30–45% of claims (1). In most social security systems, the assessment of work ability and of the resulting claim is based on a medical examination (2). While the validity and reliability of such examinations are crucial quality criteria, there is evidence for inhomogeneity of expert decisions and consequent inequality of claimants before the law (3, 4). The current use of unstructured and non-validated procedures further provokes inaccurate assessments of claimants' personal resources, potential for rehabilitation, and social and occupational outcomes (5, 6). In Switzerland, similar criticism has also arisen from within the medical community, as well as from politicians and the general public. For example, current assessment procedures have been likened to a lottery, where the quality of assessment is largely a matter of chance, due to the random assignment of claimants to experts and to alleged substantial differences in expert competence (7–10).

The insufficient reliability of psychiatric assessments has also been discussed in other German-speaking countries (11, 12), with references to the discourse in Switzerland (13) and attempts to improve education by establishing uniform quality criteria (14).

Standardization of assessments is one way to improve their homogeneity and comprehensiveness. A first step toward this goal are guidelines specifying formal criteria for structuring assessment procedures. A further gain should result from the use of standardized, structured instruments for assessing work ability (15, 16).

In German-speaking countries, the *de facto* standard tool for work ability assessment in psychiatric patients is the Mini-ICF-APP (17, 18), which contains components of the WHO's International Classification of Functioning, Disability and Health (ICF). It is, however, not validated for the insurance medical setting. As far as we know, among the many existing instruments, none are validated and specific to mental disorders in such a setting (19, 20). Thus there appears to be a lack of validated structured instruments for the expert assessment of work ability in individuals affected by mental disorders (19).

The present study is part of a project to develop such an instrument called REAcT (= “Risikoeinschätzung für Einschränkungen der beruflichen Aktivität und Teilhabe,” or, in English: “Risk assessment for restrictions of work activity and participation”). Phase one of the development consisted in an evaluation of perceived needs and difficulties in this area of testing. In particular, we were interested to see whether the lack of standardized tools would emerge as an actual need in the reports of medical professionals. Responses were collected in an online survey distributed among German-speaking therapists and medico-legal experts. Its results are reported here in a largely descriptive manner.

## Materials and methods

### Survey and sample

Survey questions were formulated based on the authors' interest, in particular the ongoing development of REAcT. The survey was hosted online by “Research Electronic Database Capture” (REDCap) (21). In June 2017, a link to the survey was distributed to German-speaking therapists and medico-legal experts (forensic psychologists and psychiatrists, and insurance physicians) via a mailing list for announcements of continuing education events, and leaflets were distributed at such events over the past 5 years. The mailing list consisted of 400, mostly Swiss and some German, recipients, including all members of the Swiss Society of Forensic Psychiatry. Initial recipients were encouraged to recruit further respondents. Data was gathered for 4 months. No systematic attempt at sampling representative sections of a larger population was made.

The survey contained 58 questions: 1 item each for age and gender, 2 items about respondents' medical certification, 2 items about continuing education/additional training, 1 item about area of work, 1 item about the number of completed professional assessments of work ability, 7 items about perceived needs for a standardized instrument for work ability assessment, 2 items about respondents' current use of standardized instruments, 1 item about the perceived difficulty of assessing work ability in different psychiatric disorders, 1 item about the estimated agreement among experts for these disorders, and 39 items about specific aspects of the REAcT questionnaire (these are not part of the present report). The questionnaire as used in this study (19 items) can be found in the Appendix at the end of the manuscript.

Bern's cantonal ethics committee filed a letter of non-competence stating no objection (Req-2017-00278).

### Statistics

Frequency data was analyzed using χ^2^-tests, while Kruskal-Wallis tests were used for comparing continuous and ordinal data across groups. The Kruskal-Wallis test is a multisample generalization of the two-sample Wilcoxon (Mann-Whitney) rank-sum test and yields the same statistical inference (*p*-value) in the two-group case. It avoids the assumption of normality of the data.

Some categorical data (Figures 2–4) is visualized using “spine plots” (22), which, like stacked bar charts, show the proportion of various categories among two or more groups, but additionally encode the number of subjects per category or group in the width of the bars. Thus, it becomes easy to see whether, for example, one group was much smaller than its comparison group, and the size of its category proportions correspondingly less trustworthy. Analyses were performed in R 3.3.3 for Mac.

## Results

### Sample

By the end of the data acquisition period, *N* = 104 responses had been collected. About two thirds of the questions had complete data, the rest had 5 missing values each. The mean age of the sample was 45 years, and 39% of respondents were women. There was no statistically significant age difference between the sexes [H(1) = 0.15, *p* = 0.22]. Sixty-Nine percent of participants were physicians (a breakdown into specialties is reported in the next section). Forty percent of participants were medico-legal experts, 55% were therapists involved with work-disabled psychiatric patients, and 5% of the data was missing. Medico-legal experts were defined as respondents with certified training in insurance-medical assessment.

The sex distribution among professional groups was almost identical: 39% of medico-legal experts were women Vs. 40% of therapists [$\chi_{\left(1\right)}^{2}$ = 0.04, *p* = 0.85].

Table 1 Lists the Characteristics of the Sample.

**Table 1:** Sample description.

	**N (%)**
Sample size	104 (100)
Missing^a^	5 (4.8)
Female	39 (39.4)
**AREA OF WORK**	
Medico-legal	42 (40.4)
Therapeutic	57 (54.8)
Missing	5 (4.8)
**N OF COMPLETED ASSESSMENTS OF WORK ABILITY**	
0–20	60 (60.6)
21–50	17 (17.2)
51–100	5 (5.1)
>100	17 (17.2)
	Mean (SD)
Age	45.4 (10.2)

a *Missing values are present in 14 (74%) of the 19 questionnaire items*.

### Certification in medical specialty

Sixty-Nine percent of the sample had a certification in some field of medical specialization. Sixty-one percent were specialized in (adult) psychiatry and psychotherapy, Two percent in child and adolescent psychiatry and psychotherapy, another Two percent in internal medicine, one percent in psychosomatic medicine and Eleven percent in other fields, including cardiology, sexual therapy, and neurology.

### Further education

Of our respondents, 42% had received further education in the area of insurance medicine; 23% had attained the title of Certified Medical Expert in Swiss Insurance Medicine (SIM); 15% had become certified forensic psychiatrists and psychotherapists SGFP (Swiss Society for Forensic Psychiatry); 5% had attained the title of Certified Medical Examiner SGV (Swiss Society of Insurance Physicians); 2% were certified physicians for social insurance medicine (“RAD physicians”) and 7% gave various other answers, each of which appeared only once.

### Area of work

In all, 49% of respondents worked in a clinic, day clinic or outpatient clinic, 34% were practitioners, 21% worked at a medical assessment center, 2% worked in an insurance company and 13% gave various other answers, only one of which occurred more than once.

### Number of assessments completed

The total number of completed assessments of work ability was less than 21 in 61% of participants, 21 to 50 in 17% of participants, 51-100 in 5%, and more than 100 in 17%. Using a somewhat arbitrary cut-off of 20 lifetime assessments, we considered a total of roughly 40% of experts “experienced.”

### Currently used instruments for work ability assessment

In their own work, 37% of respondents already used a standardized instrument for work ability assessment. A further 30% used the Mini-ICF-APP (23), 13% used the GAF (Global Assessment of Functioning scale), 2% used ICF-CorSets, and 6% reported other instruments: 1 person listed the IFAP-1(16), while all other entries referred to tests not specific to work ability assessment [e.g., HAM-D (Hamilton Scale for depression), SCL-90R (Symptom Complaint List−90 Revised), WAIS-IV (Wechsler Adult Intelligence Scale Version IV) etc].

### Need for standardized assessment instrument

**Figure 1:**
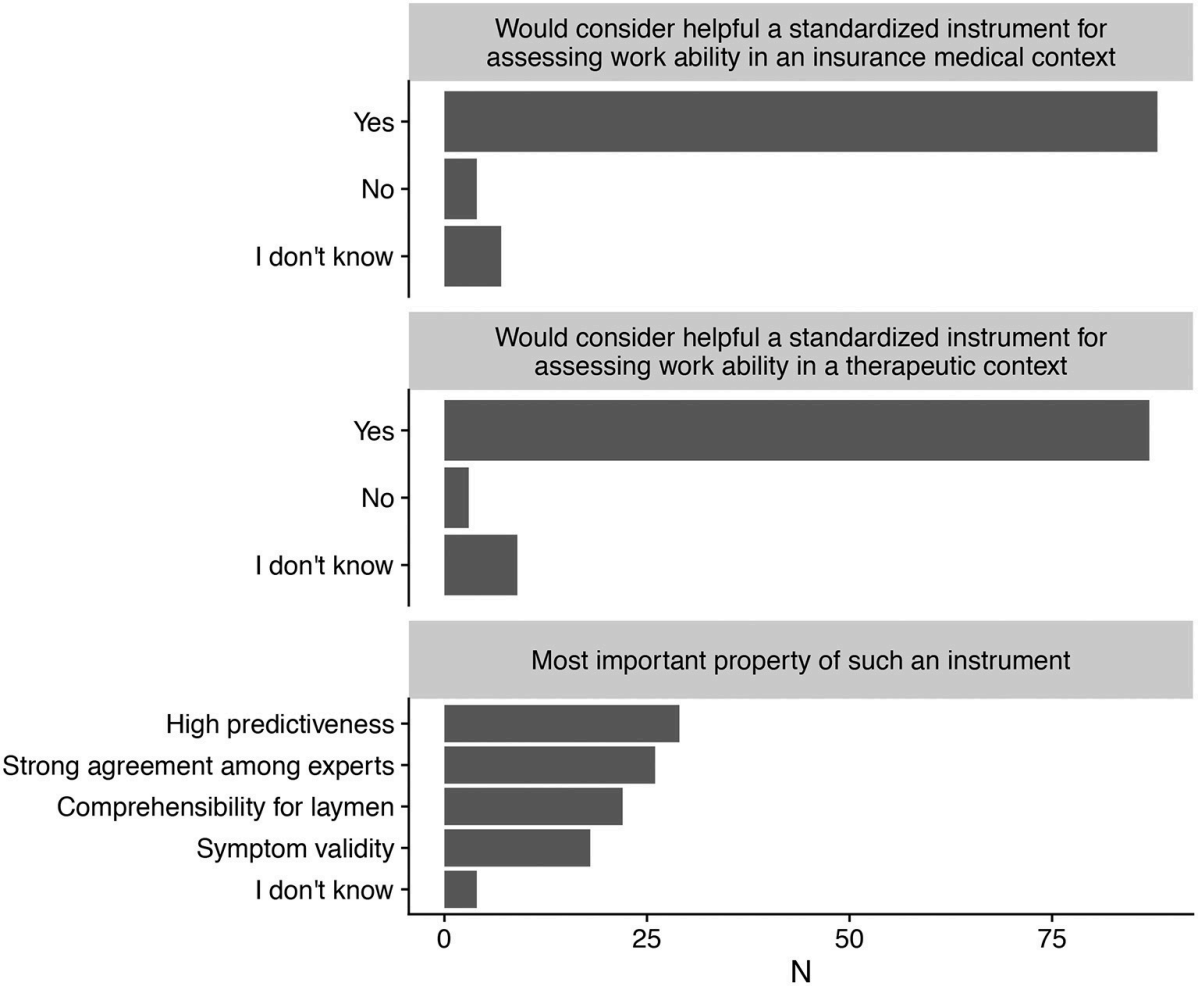
Desirability of a structured, standardized assessment of work ability in an insurance medical **(Top)** and therapeutic **(Middle)** context, as well as most important property expected of such an instrument **(Bottom)**.

Figure 1 shows that the vast majority of respondents would welcome a standardized, structured tool for work ability assessment (top two panels).

### Preferred form of instrument

Half of respondents said they would prefer an online instrument, 37% preferred a paper version, while 13% had no preference.

### Preferred type of rating

In all, 59% of respondents preferred an informant-rating scale (for use by the expert), 13% preferred a self-rating scale (for use by the patient), while 29% were uncommitted.

### Preferred property of instrument

Responses on the most important and desirable feature of such a new tool were fairly evenly distributed: predictiveness, inter-expert agreement, comprehensibility for laymen, and symptom validity all received between 20 and 30% of the responses (bottom panel).

Medico-legal experts and therapists did not differ appreciably in their responses, except perhaps for a smaller proportion of medico-legal experts emphasizing an instrument's comprehensibility for laymen.

### Importance of symptom validity tests

The most frequent view (44%) on the importance of symptom validity tests was that they should only be administered when the expert had reason to suspect that a claimant's reports on complaints might not be truthful. The view that they are always necessary was about half as frequent (23%). A surprisingly high proportion (25%) of participants expressed no opinion.

**Figure 2:**
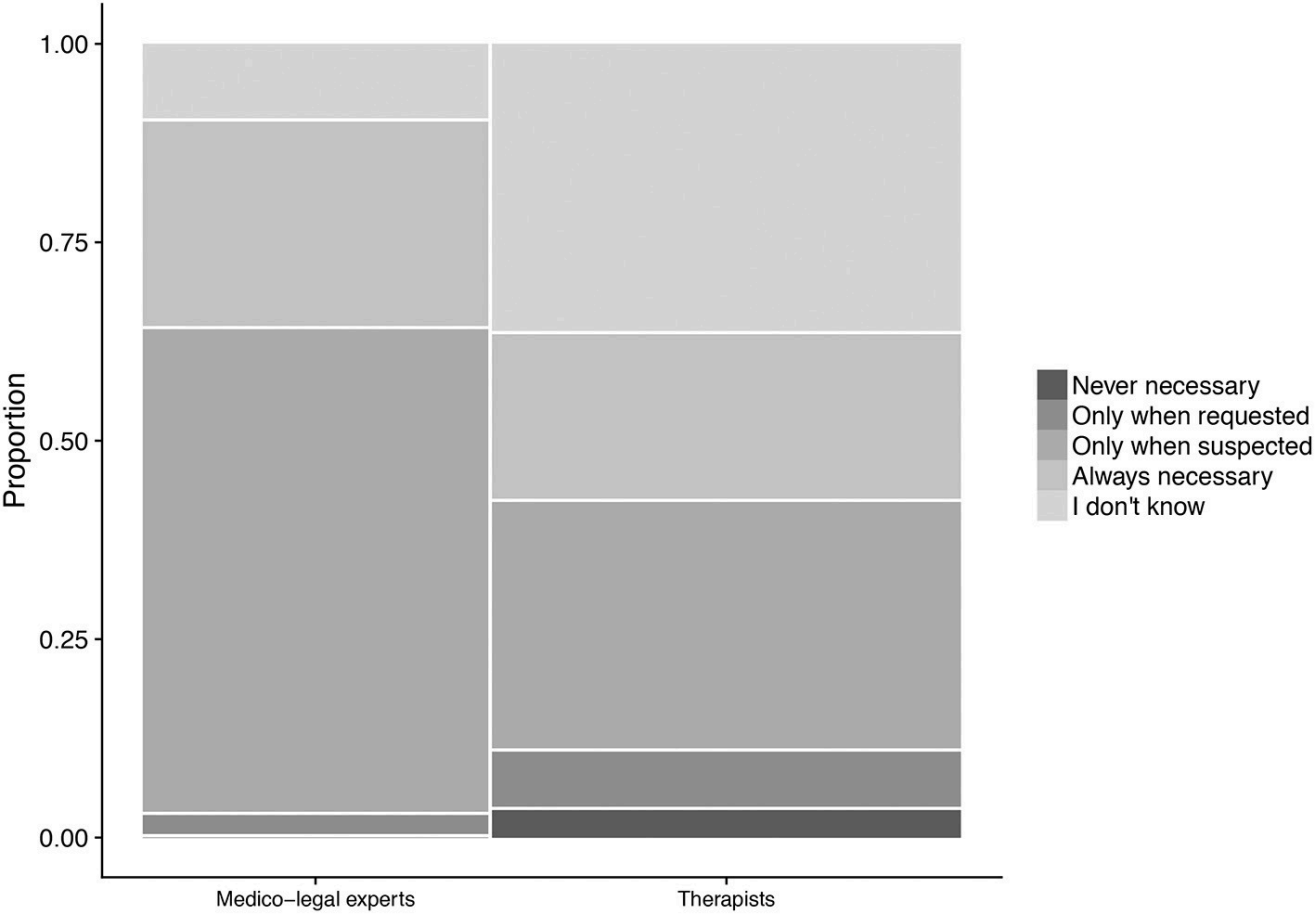
Perceived necessity of symptom validation, broken down by area of work. The width of the categories represents the frequency of the corresponding response in the total sample.

Medico-legal experts were less likely than therapists to find symptom validation never necessary (0 vs. 3.5%) or only when requested (2.4 vs. 7.0%). Also, they were much less likely to give “I don't know” as an answer (9.5 vs. 36.8%; Figure 2). The overall group test yielded χ^2^(4) = 14.93, *p* = 0.005.

Moreso than men, women favored the strict necessity of symptom validation (60.9 vs. 39.1%), whereas the small proportion (2.0%) of respondents finding it never necessary consisted exclusively of men. The overall group test yielded χ${}_{\left(4\right)}^{2}$ = 8.54, *p* = 0.07.

### Perceived difficulty of assessing various mental disorders

For each of six categories of psychiatric disorders (affective, psychotic, substance use, pain, personality, developmental), participants were asked to rate whether or not they considered it to be the most difficult disorder to assess for work ability, and how likely they considered different experts to agree on such assessments.

Respondents sometimes nominated more than one disorder as being the most difficult to assess, which led to the number of nominations exceeding the number of respondents. This was unexpected, as we assumed asking for the one most difficult among a group of disorders would elicit only one answer per person. However, given this *de facto* multiple-response format, disorders in descending order of difficulty were: pain (78 nominations), personality (63 nominations), affective (27 nominations), substance use (20 nominations), developmental (8 nominations), and psychotic (4 nominations).

### Expected expert agreement on various mental disorders

**Figure 3:**
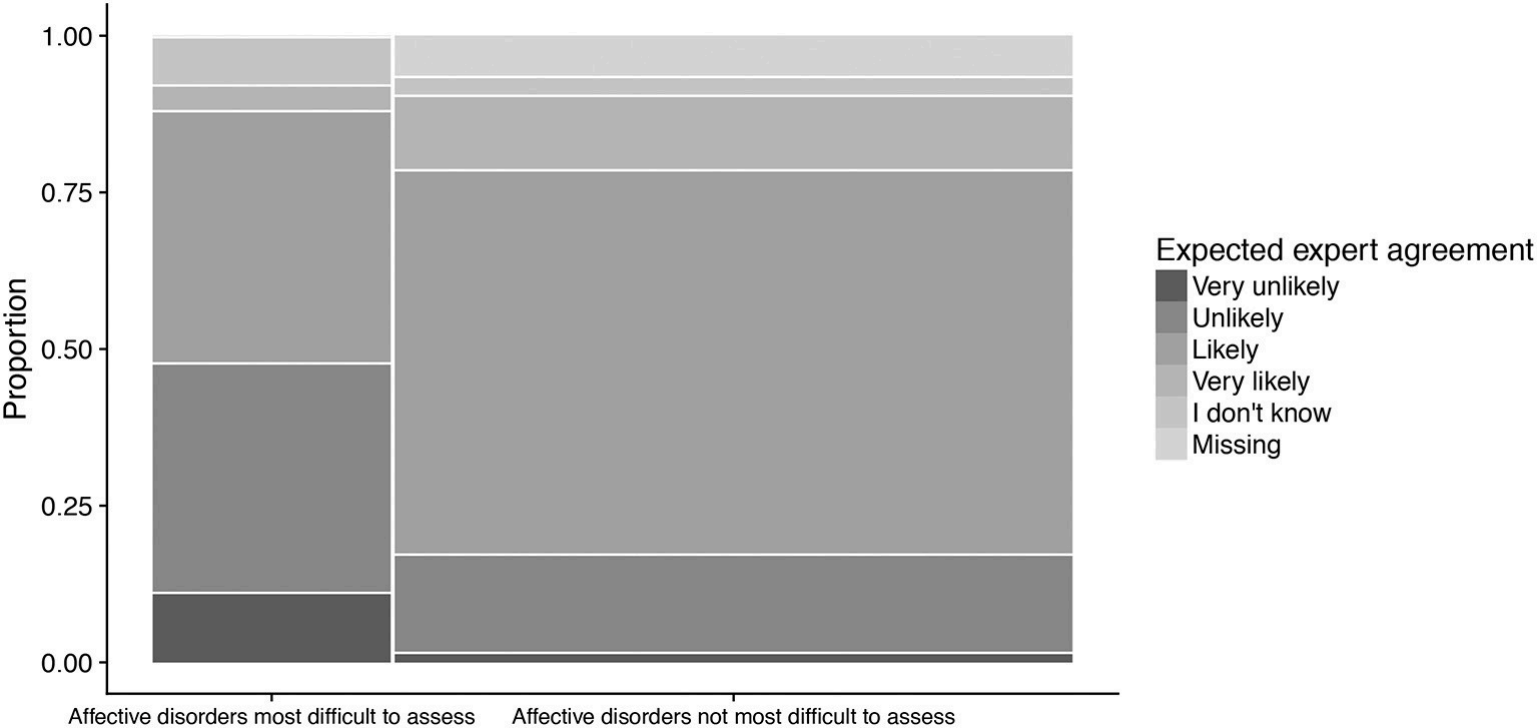
Expected expert agreement on work ability assessments of affective disorders, as a function of perceived difficulty of such assessments. Respondents considering affective disorders as most difficult to assess tended to find expert agreement more unlikely than respondents who did not. The width of the categories represents the frequency of the corresponding response in the total sample.

We found that the more a disorder was considered difficult to assess, the less respondents expected expert agreement on such assessments. This effect was clearest in the case of affective disorders (Figure 3), where the overall group test yielded χ${}_{\left(5\right)}^{2}$ = 15.28, *p* = 0.009.

**Figure 4:**
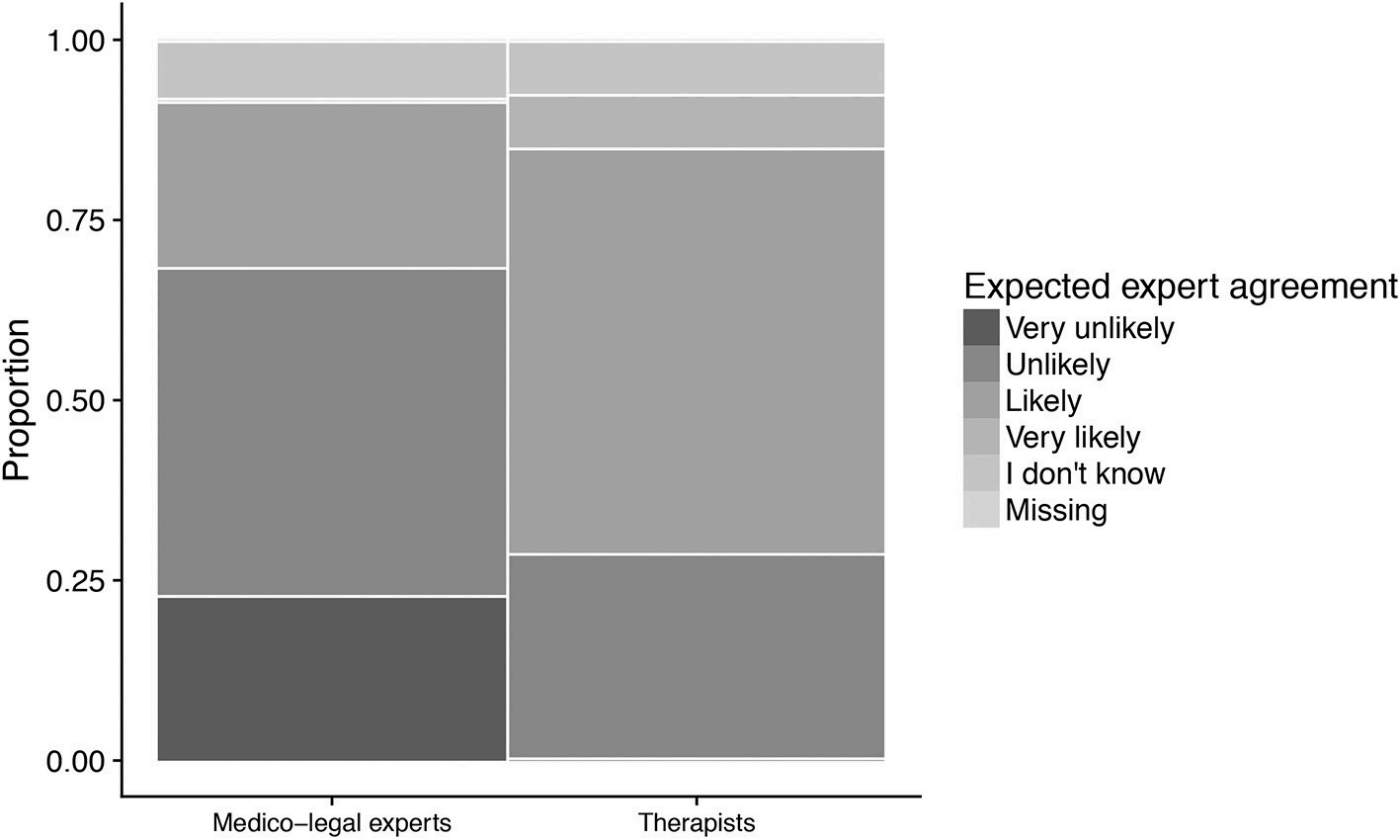
Expected expert agreement in work ability assessments for affective disorders, by area of work. Only those considering affective disorder most difficult to assess are shown (*N* = 27). Medico-legal experts tended to be more skeptical about expected agreement than therapists. The width of the categories represents the frequency of the corresponding response in the sample.

Among those finding affective disorders the most difficult to assess (*N* = 27), medico-legal experts tended to expect less agreement than therapists (Figure 4). More of them considered an agreement ‘very unlikely' (23.1 vs. 0%) or ‘unlikely' (46.2 vs. 28.6%), while comparatively fewer expected agreement to be “likely” (23.1 vs. 57.1%) or “very likely” (0 vs. 7.1%). The overall group test yielded $\chi_{\left(4\right)}^{2}$ = 6.64, *p* = 0.16.

Men tended to expect less expert agreement than women (15.8 vs. 0% for “very unlikely,” 0 vs. 12.5% for “very likely”). The overall group test yielded $\chi_{\left(4\right)}^{2}$ = 4.07, *p* = 0.40. However, due to the small sample size, these test result can be assumed to be somewhat unreliable.

## Discussion

Our survey among 104 German-speaking professionals documented the wish for a standardized, structured instrument to assess work ability. Respondents were overwhelmingly in favor of such a tool both in an insurance medical and therapeutic setting. However, roughly equal proportions (~20–30%) of participants named four characteristics as the most desirable property of the instrument: high predictiveness, high inter-rater agreement, high comprehensibility for laymen, and high symptom validity. While this flat distribution may reflect a diversity of informed opinion, it may also be the case (as one reviewer suggested) that these concepts were somewhat foreign to respondents, introducing unsystematic variation into their answers.

Symptom validity, the truthfulness of a claimant's report of complaints, was rated as more important by women than men, and by medico-legal experts compared with therapists. The explanation of the sex difference, if it is a real effect, is not obvious to us. One plausible idea is that the higher levels of conscientiousness found in women (24) may make them consider it more of a duty (than men would) to first rule out malingering before proceeding further. The finding regarding medico-legal experts, on the other hand, may simply reflect the perception that malingering is more frequent in a medico-legal than in a clinical setting (25). Further, there might have been a lack of understanding of the concept of symptom validity in therapists, contributing to lower importance ratings. In any case, the between-group heterogeneity in importance given to symptom validation should be considered in the development of the REAcT and other instruments, e.g., by instructing users to pay special attention to symptom validation in order to achieve robustly high rates of this practice across all groups.

Asking respondents about which of six categories of psychiatric disorders was most difficult to assess for work ability yielded the following ranking by decreasing difficulty: pain disorder, personality disorders, affective disorders, substance use disorders, developmental disorders, and psychosis. This rank order is partly reflected in Swiss federal court rulings, which are more complex for pain and affective disorders and involve special legislation (26).

Not surprisingly, more difficult disorders were expected by participants to lead to lower expert agreement in assessing work ability. This effect was most pronounced for affective disorders. This finding is consistent with the majority of participants wishing for a standardized and structured assessment tool, as such a tool would be expected to reduce assessment difficulty and consequently lead to higher inter-rater agreement, i.e., higher reliability.

Our study had a number of limitations: First, the sample was small and statistical power correspondingly low. Second, due to the non-random distribution of the survey, including snowball sampling, it is not known to what extent current results are generalizable to a larger population. Third, we had no control over possibly untruthful survey responses.

In conclusion, the results of our survey document professionals' wish for a structured, standardized instrument to assess work ability in both insurance medical and therapeutic settings. This study was the first part of a project that aims at the development of such an instrument.

## Author contributions

All authors were responsible for conceiving and designing the study and contributed to data interpretation. AG and IW conducted the data analysis. RS and ML were responsible for data acquisition and contributed to its interpretation. RS, AG, and ML wrote the manuscript, and all co-authors revised its content. All authors approved the final version of the manuscript and agree to be held accountable for all aspects of the work.

### Conflict of interest statement

WR has been the chief editor of the present journal (Frontiers in Psychiatry–Public Mental Health) for the past 4 years. He was not involved in any aspect of the processing of this manuscript. The remaining authors declare that the research was conducted in the absence of any commercial or financial relationships that could be construed as a potential conflict of interest.
