# Supplementary material for: Online Survey of Medical and Psychological Professionals on Structured Instruments for the Assessment of Work Ability in Psychiatric Patients
Source: Front Psychiatry. 2018 Sep 25;9:453. doi: 10.3389/fpsyt.2018.00453 (PMC6167551; doi:10.3389/fpsyt.2018.00453)
Supplement: Supplementary file 1 [file Data_Sheet_1.docx]

**Appendix: Questionnaire items**

1. Age [years]
2. Sex
   1. Male
   2. Female
3. Do you have one or more certifications in any medical specialty?
   1. Yes
   2. No
4. What is your certification?
   1. Internal Medicine
   2. Child or Adolescent Psychiatry or Psychotherapy
   3. Psychiatry or Psychotherapy
   4. Psychosomatic Medicine
   5. Other: Please specify
5. Have you completed additional training in the area of insurance medicine/assessment?
   1. Yes
   2. No
6. Which additional training have you completed?
   1. Certified Medical Expert Swiss Insurance Medicine (SIM)
   2. Certified Medical Examiner SGV
   3. Certified physician for social insurance medicine ("RAD physicians")
   4. Master in Insurance Medicine (MASIM)
   5. Certified work ability assessor (ZAFAS)
   6. Certified forensic psychiatrist and psychotherapist SGFP
   7. Other: Please specify
7. In which area do you work?
   1. Private Practice
   2. Clinic, day clinic, outpatient clinic
   3. Medical assessment center
   4. Insurance
   5. Other: Please specify
8. Number of total insurance-medical assessments of work ability?
   1. 0 – 20
   2. 21 - 50
   3. 51 - 100
   4. > 100
9. Would you find a standardized instrument for assessing work ability in an insurance medical context helpful?
   1. Yes
   2. No
   3. I don't know
10. Would you find a standardized instrument for assessing ability to work in a therapeutic context helpful?
    1. Yes
    2. No
    3. I don't know
11. Which form of application for such an instrument would you prefer?
    1. Online
    2. Paper
    3. I don't know
12. Do you already use standardized instruments for assessing ability to work?
    1. Yes
    2. No
13. If so, which instrument(s) do you use?
    1. Mini-ICF-APP
    2. ICF-CorSets
    3. GAF
    4. Other: Please specify
14. Which kind of instrument would you prefer in a therapeutic context?
    1. Self-rating scale (completed by patient)
    2. Informant-rating scale (completed by expert)
    3. I don't know
15. Which kind of instrument would you prefer in an insurance-medical context?
    1. Self-rating scale (completed by patient)
    2. Informant-rating scale (completed by expert)
    3. I don't know
16. Which property of an instrument for assessing work ability is most important to you?
    1. Symptom validity
    2. Strong agreement among experts
    3. High predictiveness"
    4. Comprehensibility for laymen
    5. I don't know
17. What is your opinion on the use of symptom validation tests in work ability assessments?
    1. Their use is never necessary
    2. Their use is only necessary when officially requested
    3. Their use is only necessary when a claimant's lack of truthfulness is suspected
    4. Their use is always necessary
    5. I don't know
18. Which psychiatric disorders pose the greatest difficulties for assessing work ability?
    1. Affective disorders
    2. Psychoses
    3. Substance use disorders
    4. Pain or somatoform disorders
    5. Personality disorders
    6. Developmental disorders
    7. Other: Please specify
19. How likely do you consider expert agreement on the assessment of work ability for said disorders?
    1. Affective disorders
    2. Psychoses
    3. Substance use disorders
    4. Pain or somatoform disorders
    5. Personality disorders
    6. Developmental disorders

Response options for a – f:

- Very unlikely
- Unlikely
- Likely
- Very likely
- I don't know
